# MTHFD2- a new twist?

**DOI:** 10.18632/oncotarget.7147

**Published:** 2016-02-03

**Authors:** Philip M. Tedeschi, Kathleen W. Scotto, John Kerrigan, Joseph R. Bertino

**Affiliations:** Departments of Pharmacology and Medicine, Robert Wood Johnson Medical School and Rutgers Cancer institute of New Jersey, New Brunswick, NJ, USA

**Keywords:** one carbon (folate) metabolism, serine metabolism, glycine metabolism, MTHFD2, anti-folate

**Figure 1:**
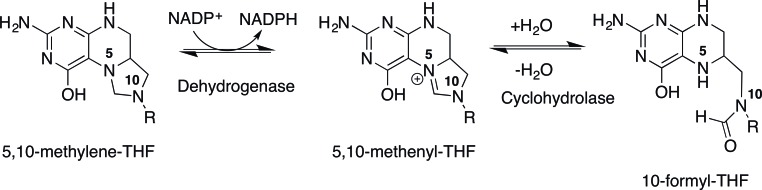
The cytoplasmic enzyme, MTHFD1, uses NADP as a cofactor as compared to MTHFD2, which carries out the same enzyme activity using NAD, Mg++ and PO4-. R= p-aminobenzoylglutamate

Rapidly proliferating tumors attempt to meet the demands for nucleotide biosynthesis by up-regulating folate pathways that provide the building blocks for pyrimidine and purine biosynthesis. Reduced folates are carriers of one carbon units required for the synthesis of purines, thymidylate and methionine, derived from serine, glycine and formate. As folate metabolism plays a key role in cell proliferation, the folate-requiring enzymes dihydrofolate reductase and thymidylate synthase have long been key targets for treatment of cancer. Recent studies show that the mitochondrial folate enzymes are also critical, in that they enable mitochondria to produce additional one carbon units for purine synthesis to allow for rapid growth. In transformed cells, methylene tetrahydrofolate dehydrogenase MTHFD2 is often reactivated and expressed along with other members of the serine synthesis, one carbon (folate) metabolism and glycine cleavage system, allowing for enhanced production of purines, ATP and NADPH, fueling cell proliferation [1]. More recently, it has been recognized that these enzymes are critical for the generation of NADH/NADPH, necessary for protection from ROS and required for macromolecular synthesis. MTHFD2 is a bifunctional enzyme with methylene dehydrogenase and cyclohydrolase activity that produces N-10 formyl tetrahydrofolate, the source of C2 and C8 in purines and NADH from methylenetetrahydrofolate and NAD [2]. The cytoplasmic enzyme, MTHFD1 uses NADP as a cofactor as compared to MTHFD2, which carries out the same enzyme activity using NAD, Mg++ and PO4-. In rapidly growing cancer cells, but not normal proliferating cells, MTHFD2 is the major source of formate for purine synthesis (Figure 1).

Using gene expression arrays, we have shown that overexpression of mitochondrial enzymes, particularly MTHFD2, is associated with both high proliferation rates and cMYC overexpression [3]; this key role for MTHFD2 in cancer cell proliferation has recently been confirmed [4]. Most importantly, overexpression of MTHFD2 has been shown to be associated with poor prognosis of patients with breast cancer [5] and with an increased rate of invasion and metastasis [6]. That MTHFD2: 1) is over expressed in rapidly replicating tumor cells but not in adult tissue, and 2) enhances tumor cell proliferation provides a strong rationale for targeting this enzyme for selective cancer treatment [7].

The New Twist. It has recently been shown that MTHFD2 can have an impact on proliferation independent of its enzymatic activity [8]. In these studies, MTHFD2 was found in the nucleus, and co-localized with DNA replication sites. How this interaction enhances proliferation is unknown. That MTHFD2 has a dual effect on tumor cell proliferation, i.e., enhancing nucleotide synthesis directly and possibly “moonlighting” as a DNA binding protein [8] makes it an even more important and selective target for cancer treatment, but suggests that inhibition of enzyme activity alone may not be sufficient to effect tumor regression. If inhibition of this enzyme activity proves to be not effective, new approaches targeting transcription or translation may be required to achieve anti-tumor activity.
